# Serum trace metal association with response to erythropoiesis stimulating agents in incident and prevalent hemodialysis patients

**DOI:** 10.1038/s41598-020-77311-8

**Published:** 2020-11-19

**Authors:** Michael E. Brier, Jessica R. Gooding, James M. Harrington, Jason P. Burgess, Susan L. McRitchie, Xiaolan Zhang, Brad H. Rovin, Jon B. Klein, Jonathan Himmelfarb, Susan J. Sumner, Michael L. Merchant

**Affiliations:** 1 Kidney Disease Program, Department of Medicine, University of Louisville, Louisville, KY 40202 USA; 2 The Robley Rex Veterans Affairs Medical Center, Louisville, KY 40206 USA; 3 NIH Eastern Regional Comprehensive Metabolomics Resource Core, RTI International, Research Triangle Park, NC 27709 USA; 4 NIH Common Fund Eastern Regional Comprehensive Metabolomics Resource Core (ERCMRC) and Nutrition Research Institute, University of North Carolina, Chapel Hill, NC 28081 USA; 5 Analytical Sciences Department, RTI International, Research Triangle Park, NC 27709 USA; 6 Department of Internal Medicine, Ohio State University, Columbus, OH 43210 USA; 7 Department of Medicine, Kidney Research Institute, University of Washington, Seattle, WA 98195 USA; 8 Center for Integrated Environmental Health Sciences, University of Louisville, Louisville, KY 40202 USA

**Keywords:** Environmental impact, Haemodialysis

## Abstract

Alterations in hemodialysis patients’ serum trace metals have been documented. Early studies addressing associations levels of serum trace metals with erythropoietic responses and/or hematocrit generated mixed results. These studies were conducted prior to current approaches for erythropoiesis stimulating agent (ESA) drug dosing guidelines or without consideration of inflammation markers (e.g. hepcidin) important for regulation of iron availability. This study sought to determine if the serum trace metal concentrations of incident or chronic hemodialysis patients associated with the observed ESA response variability and with consideration to ESA dose response, hepcidin, and high sensitivity C-reactive protein levels. Inductively-coupled plasma-mass spectrometry was used to measure 14 serum trace metals in 29 incident and 79 prevalent dialysis patients recruited prospectively. We compared these data to three measures of ESA dose response, sex, and dialysis incidence versus dialysis prevalence. Hemoglobin was negatively associated with ESA dose and cadmium while positively associated with antimony, arsenic and lead. ESA dose was negatively associated with achieved hemoglobin and vanadium while positively associated with arsenic. ESA response was positively associated with arsenic. Vanadium, nickel, cadmium, and tin were increased in prevalent patients. Manganese was increased in incident patients. Vanadium, nickel, and arsenic increased with time on dialysis while manganese decreased. Changes in vanadium and manganese were largest and appeared to have some effect on anemia. Incident and prevalent patients’ chromium and antimony levels exceeded established accepted upper limits of normal.

## Introduction

Hemodialysis (HD) is a blood purification method intended to remove metabolic waste products (‘uremic toxins’) by primarily equilibrating the composition between the patients sera (higher concentrations) and the dialysate (lower concentrations) across a semipermeable dialyzer membrane^1^. In practice the method removes both low and medium molecular weight compounds including normal biologics as well as uremic toxins. Many factors affect the equilibration of solutes across the dialyzer membrane including concentration, size, charge, and compartmentalization. Chronic hemodialysis as a medical tool has been used as supportive care for individuals who have lost the function of their kidneys. The largest fraction of dialysis care is provided to adults 65 years old or greater and in this population HD is associated with an average life expectancy of 5 years^2^. Multiple co-morbid conditions contribute to the reduced life expectancy including infection, cardiovascular disease, hypertension, hyperlipidemia, diabetes, and anemia. Protein energy wasting is a prevalent finding in maintenance hemodialysis patents and may be associated with diminished levels of iron, selenium, vitamin C and other micronutrients. These data suggest that micronutrient loss including trace metals may contribute significantly to hemodialysis patient comorbidities and mortality. Thus we hypothesize an improved understanding of associations between trace metals and the anemia management may support identification of salutatory trace metals supporting a longer and an improved quality of life^3–8^.

HD patients are medically complex with many suffering from metabolic syndrome, diabetes and dyslipidemia while others suffering from protein-energy wasting syndrome, loss of appetite and reduced intake of nutrients such as trace minerals^9^. The goal of HD is to clear low molecular weight uremic toxins but it also affects beneficial low molecular weight substances including amino acids, salts, vitamins and serum trace minerals^10^. In fact, clearance of non-protein bound, low molecular weight solutes tends to be enhanced unless specifically supplemented into the dialysate fluid. Although the generation of water for dialysate purposes is heavily regulated to minimize microbial toxins, the potential still remains for trace metal contamination resulting in toxicity to the HD patients^11–14^. An example would be aluminum toxicity associated with contamination of dialysate water and/or oral phosphate binders resulting in softening of the bones (osteomalacia), anemia, or dialysis encephalopathy in hemodialysis patients^14–16^.

A meta-analysis of 128 studies on the trace element status of hemodialysis patients identified levels of cadmium, chromium, copper, lead and vanadium higher in hemodialysis patients than compared to normal controls^17^. The levels of selenium, zinc, and manganese were lower in hemodialysis patients than controls. These same authors recently reported on 1278 patients with 2-year prospective follow up measuring 25 trace elements and outcomes including death, cardiovascular events, systemic infection and hospitalization. Cardiovascular disease, the main cause of mortality in hemodialysis patients, was associated with higher zinc levels at 2 years of hemodialysis. Lower concentrations of selenium were independently associated with high risk of cardiovascular disease, all-cause hospitalization or death^3^. In an exploratory analysis, copper and cadmium concentrations were associated with a higher risk of death. Ari et al. report a strong positive correlation between high concentration of cadmium, lead, magnesium, and copper-to-zinc ratio with increased carotid artery intima-media thickness and hence with carotid artery atherosclerosis^18^.

Studies on serum trace metal levels affected by applied dialytic modality have yielded mixed results. Cross and Davenport reported no difference in observed serum zinc and selenium concentrations for maintenance hemofiltration patients that cross-over to hemodialysis^19^. However Prodanchuk et al. compared 17 serum trace metal concentrations in existing hemodialysis and hemofiltration patients and documented significantly higher serum levels of cadmium, cobalt, copper, and lead in hemodialysis patient serum samples^20^. A meta-analysis of clinical trials for the replacement of therapeutically low trace metals such as zinc via dietary supplementation has shown beneficial results. Zinc supplementation positively affected serum zinc levels as well as dietary protein intake, increased levels of the antioxidant superoxide dismutase, and decreased inflammatory markers such as C-reactive protein (CRP)^21,22^.

The kidney is responsible for the production and releasing of erythropoietin (EPO) as a function of the blood oxygen levels. The anemia of chronic kidney disease and ESKD may be associated with failed EPO production, diminished sensitivity to exogenous EPO or compounding effects of dysregulated trace metal metabolism. In the absence of the sufficient kidney function these minerals build-up in the serum as a function of dietary intake and consumption or through environmental exposure. Studies on trace metals, such as iron, copper, cobalt and zinc play essential roles in many facets of biology relevant to the complications and comorbidities associated with hemodialysis including the function of the hypoxia inducible factor-1 (HIF-1) pathway^23,24^ and erythropoiesis^25,26^. Trace metals such as manganese, zinc, chromium, and selenium can interact with and regulate iron available for erythropoiesis at the levels of iron uptake, transport, and iron storage regulatory proteins^27^. Experimentally trace metal (iron, copper, zinc, cobalt, manganese, cadmium or lead) intake or trace metal interactions with cognate-iron binding partners have been demonstrated to modify globin gene synthesis, erythrocyte production and/or metabolically reprogramming cellular differentiation pathways^27–30^. Lastly, zinc (or copper, manganese, cobalt) supplementation may have a direct (progenitor cell differentiation) or indirectly to enhance iron availability by reduction of inflammatory modulators (CRP) of red blood cell productions (erythropoiesis)^31–34^.

Central to the current discussion of anemia management with EPO and biosimilar drugs is the identification of those patients that respond normally to pharmacologic concentrations of EPO or erythropoiesis stimulating agents (ESAs) and those patients that do not. Many patients respond well to low doses of drug while other patients may require much larger doses^35–37^. Patients that are hypo-responsive to EPO may be at risk for increased morbidity and mortality^38^. In the clinical setting of ESKD and hemodialysis there are a multitude of factors which may contribute to anemia and hypo-responsiveness to ESA including inadequate ESA dose, iron deficiency, blood loss, infection, inflammation, secondary hyperparathyroidism, hemolysis, malignancies, hematologic disorders, acquired immune-deficiency syndrome (AIDS), pregnancy, vitamin deficiency, and aluminum toxicity. The critical central role that iron maintains in erythropoiesis itself may be modified by relative concentrations of other trace metals. However other trace metal differences may exist in patients with poor response to ESAs that may be detectable in the serum.

HD may contribute to trace metal dysregulation directly via loss or gain by exchange with the dialysate. Erythropoiesis requires iron and other trace metals as important co-factors in many cellular erythropoietic processes including globin gene synthesis and maturation. As such we hypothesized that ESA responsiveness would be impacted by differing concentrations of metals. We studied both incident and prevalent patients on hemodialysis from a single dialysis provider over a six-month period of time and measured ESA dose, hemoglobin, critical markers of inflammation, and 14 trace metals. Our primary objective was to analyze the relationship between ESA response and trace metal concentration. Our secondary objectives were to examine the changes in trace metal concentration between incident and prevalent patients, and between male and female given known baseline sex-dependent differences in Hb levels^39–41^.

## Results

**Figure 1:**
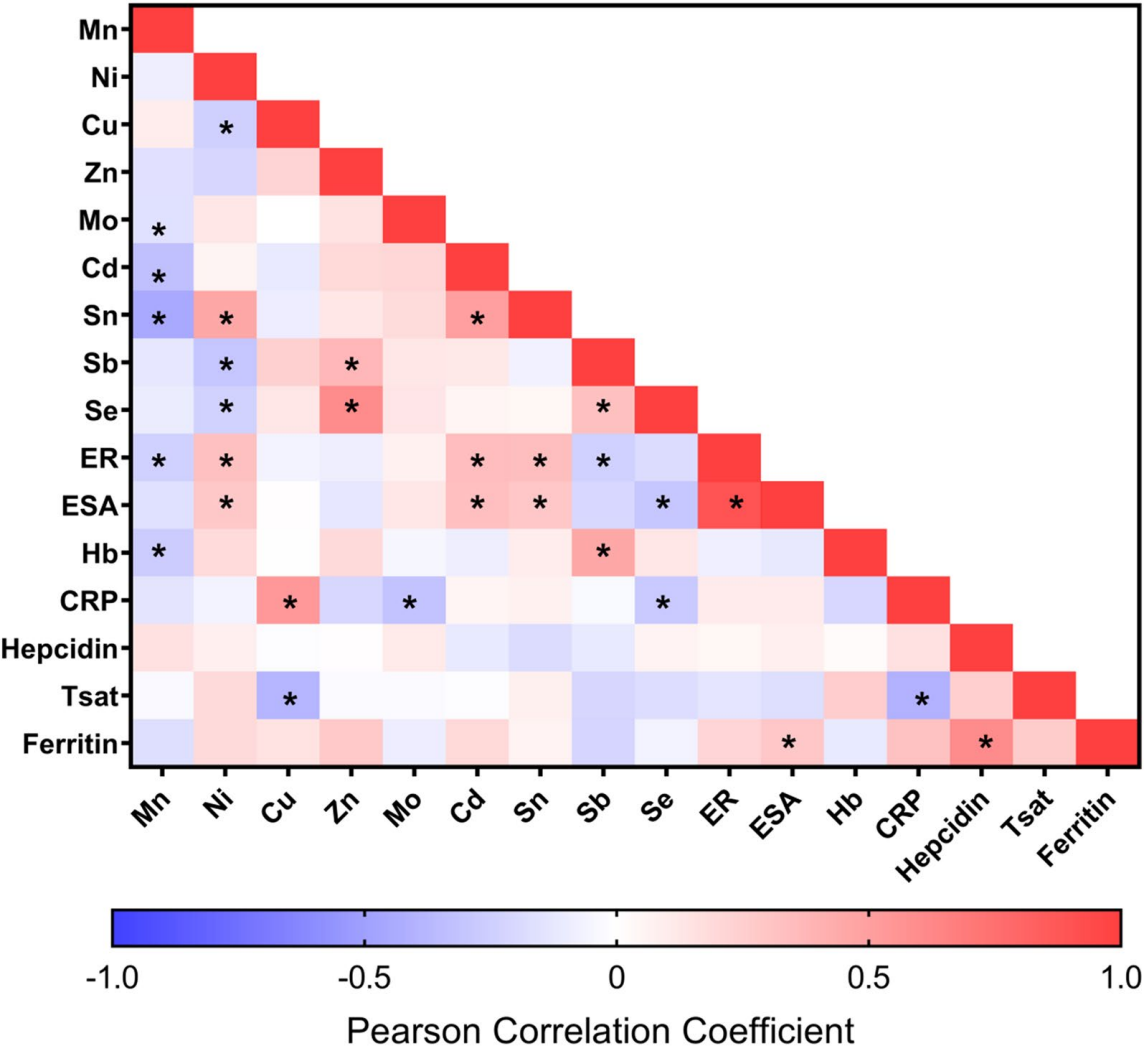
Pearson correlation between all measured variables. Red squares represent positive correlations and blue squares represent negative correlations. Asterisk (*) denotes correlation values with a p value < 0.05. Trace metal symbol definitions provided in methods.

A total of 100 prevalent and 33 incident subjects were enrolled and sufficient sample was available for analysis in 108 subjects (79 prevalent and 29 incident patients). The results for primary metals measurements have been uploaded into the metabolomics workbench of the NIH Common Fund Metabolomics Program Data Repository and Coordinating Center (DRCC) as study ST000565. The demographic information on the population are shown in Table 1. A Pearson bivariate correlation analysis is shown in Fig. 1. Significant positive correlations were observed between ER (erythropoietin response, dose/Hb achieved) and nickel, cadmium, tin; ESA dose and nickel, cadmium, tin; hemoglobin (Hb) and antimony; CRP and copper. Significant negative correlations were observed between ER and manganese, antimony; ESA dose and selenium; Hb and manganese, CRP and molybdenum, selenium; transferrin saturation (Tsat) and copper. Hepcidin was not found to be significantly correlated to any measured trace metal.

**Table 1:** Demographic data on subjects.

	Total population (n = 133)	Analysis population (n = 108)
Sex (M/F)	81 (61%) 52 (39%)	68 (63%) 40 (37%)
Race*		
African American	22	14
Caucasian	91	77
Asian American	12	10
Other	8	7
Dialysis vintage, months, mean (range)	28.7 (1, 197)	27.5 (1, 197)
Hemoglobin, g/dL, mean (range)	10.7 (7.3, 15.7)	10.7 (7.3, 15.7)
Tsat %, mean (range)	34.8 (11, 84)	30.1 (11, 84)
Ferritin mean, µg/L (range)	1113 (118, 2544)	959 (118, 2544)
CRP mean, mg/L (range)	14.2 (0, 131)	12.8 (0, 108)
Hepcidin mean, ng/mL (range)	160 (9, 594)	174 (9, 594)
EPO (epoetin alfa) (month) mean, U (range)	37,218 (0, 935,000)	28,904 (0, 136,200)

*Consistent with the patient distribution within the Northwest Kidney Centers, Seattle, WA.

**Figure 2:**
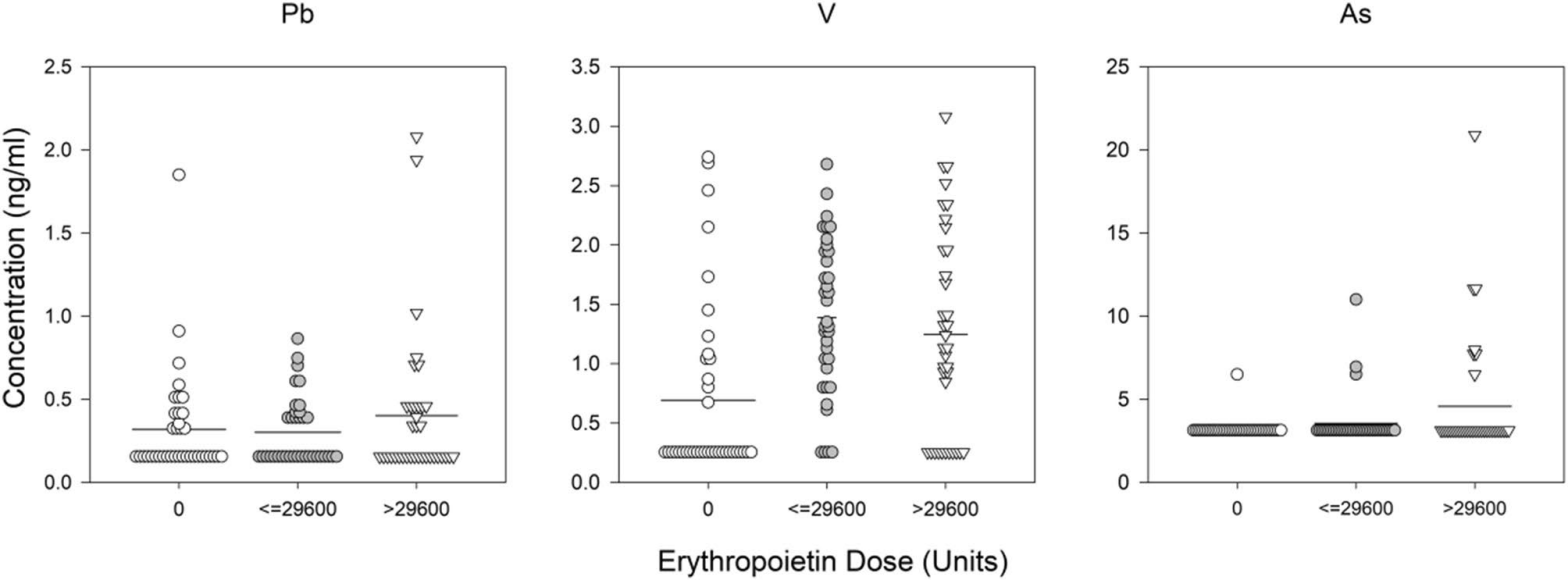
Trace metals associated with prior month ESA dose divided into categories: patients that received no ESA and those below and above the median non-zero value. Solid line represents the mean value. Trace metal symbol definitions provided in methods.

**Figure 3:**
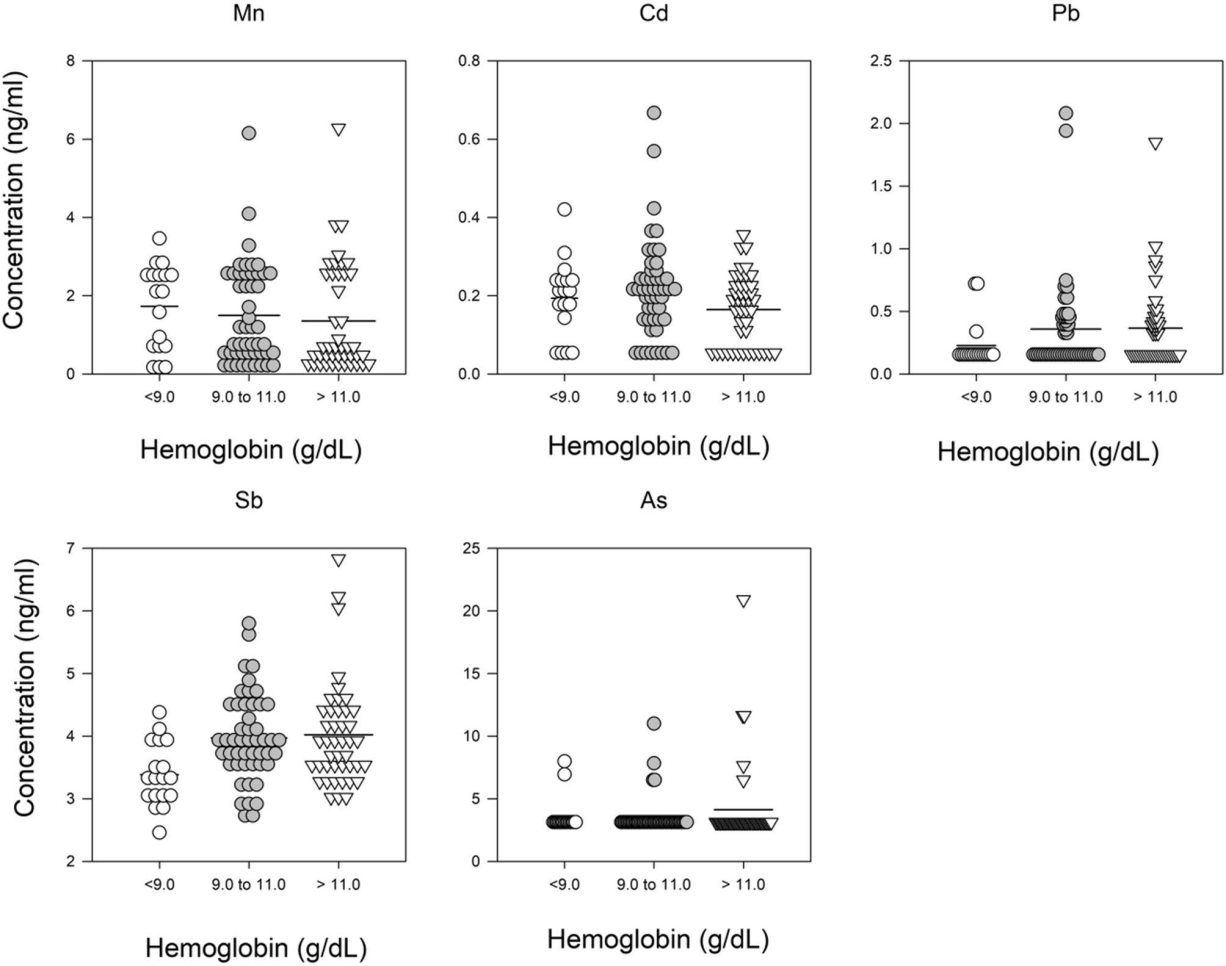
Trace metals associated with current Hb divided into categories: below 9.0 g/dL, between 9.0 and 11.0 g/dL and > 11.0 g/dL. Solid line represents the mean value. Trace metal symbol definitions provided in methods.

**Figure 4:**
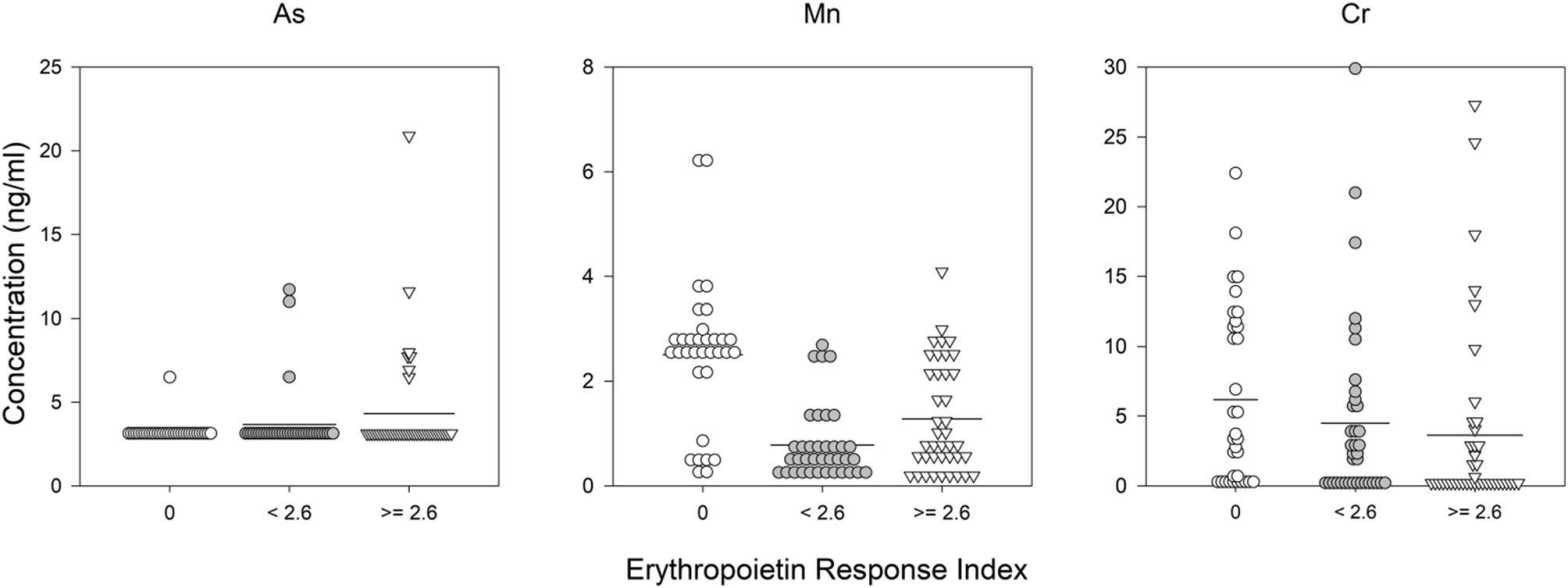
Trace metals associated with ER divided into categories: 0, did not receive an ESA for 6 months, below and greater than the median non-zero value. Solid line represents the mean value. Trace metal symbol definitions provided in methods.

To determine if there were a linear relationship of trace metals and anemia metrics, a multivariate regression of prior month ESA dose, Hb concentration and 6-month average ER and all of the trace metal concentrations was conducted. Starting with all trace metals, the final regression model was determined after sequentially removing individual trace metals that did not contribute significantly. The model results are presented in Figs. 2, 3 and 4 and Table 2 showing the variables (final trace metals) with the standardized beta coefficient demonstrating the strength of the association. The final regression model for a trace metal association with the prior month ESA dose arrived at a four variable model. The model included positive associations with arsenic (approaching significance at p-value < 0.06) and lead, and negative associations with Hb concentration and vanadium. The regression model for trace metal association with Hb concentration arrived at a six variable model that included positive associations with antimony (approaching significance at p-value < 0.08), arsenic and lead and, negatively associations with manganese, cadmium, and ESA dose. The regression model for trace metal association with ER arrived at a three component model with positive associations with manganese and arsenic, and a negative associate with chromium.

**Table 2:** Results of the regression analysis of metal concentration, sex, Hb, CRP (median), hepcidin (median) vs. prior-month ESA dose, hemoglobin in prevalent dialysis patients, or ER averaged over prior 6 months.

Model	Beta (standardized)	p value
**Prior month ESA dose**		
Constant		< 0.001
Hb concentration	− 0.362	0.001
Log2As	0.249	0.016
Log2V	− 0.223	0.032
Log2Pb	0.194	0.056
**Hemoglobin concentration**		
Constant		0.003
Log2Mn	− 0.191	0.075
Log2Cd	− 0.340	0.001
Log2Sb	0.273	0.008
Log2As	0.220	0.032
Log2Pb	0.242	0.020
ESA Dose	− 0.321	0.003
**ER**		
Constant		0.243
Log2Mn	0.187	0.099
Log2As	0.239	0.034
Log2Cr	− 0.189	0.090

Trace metal symbol definitions provided in methods.

**Figure 5:**
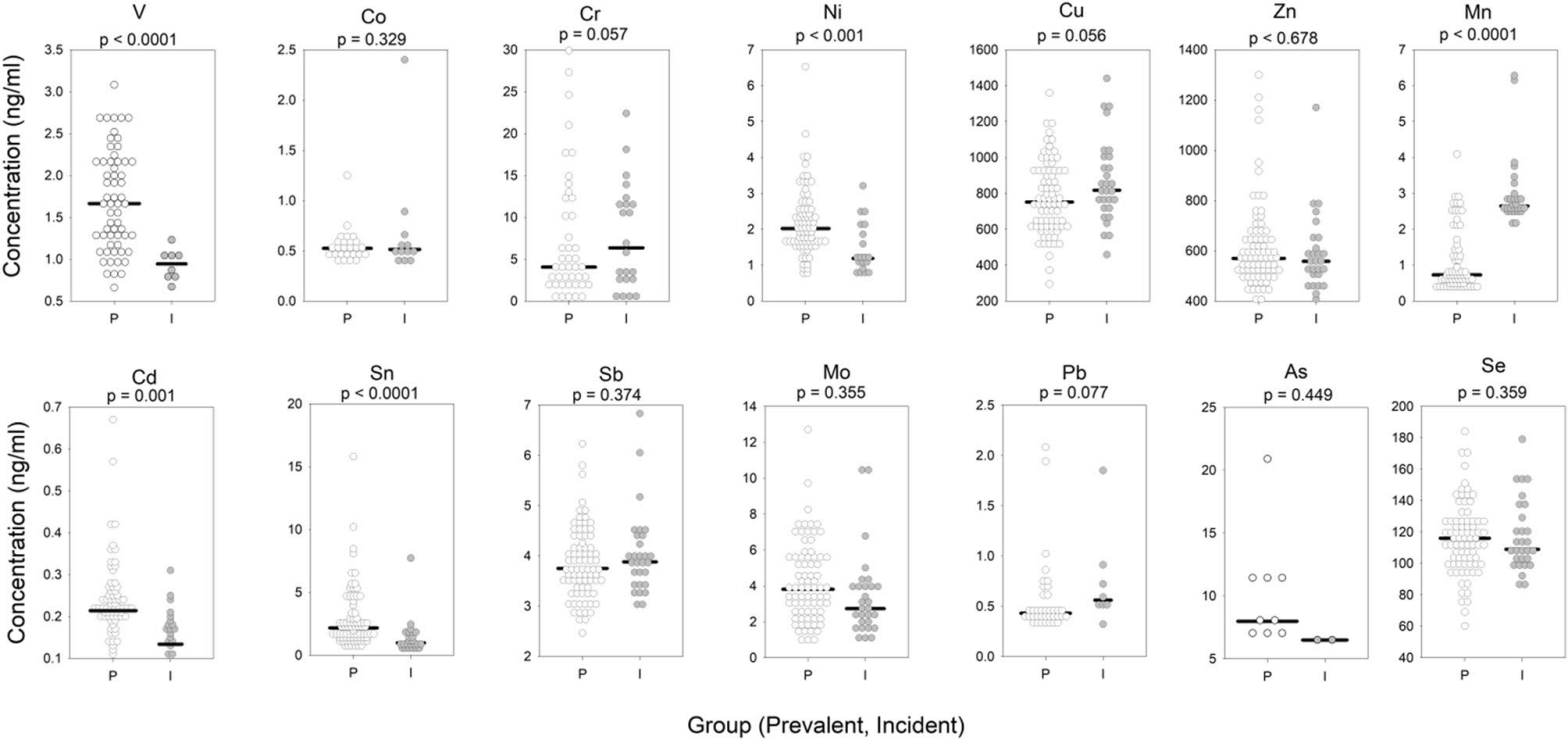
Dot density plot of the trace metal concentration in prevalent and incident patients with p values. Solid line represents the mean. Trace metal symbol definitions provided in methods.

**Figure 6:**
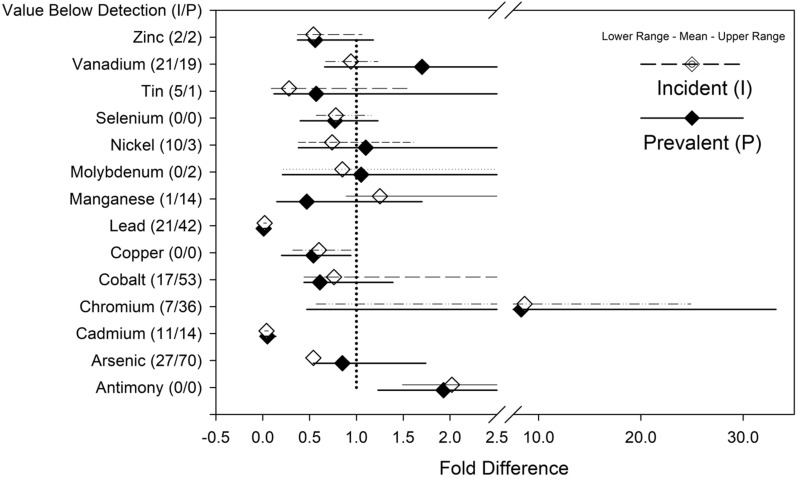
Comparison of trace metal levels in incident (n = 29) versus prevalent (n = 79) patient plasma samples as a mean fold-difference against literature value for upper limit of normal.

The results of the inductively coupled plasma-mass spectrometry (ICP-MS) analysis for each measured metal in prevalent and incident subjects are shown in Fig. 5. The differences with p-values less than 0.05 were observed for cadmium, manganese, tin, nickel, and vanadium. A linear regression of the metal concentration versus the time on dialysis showed an increase in vanadium (r2 = 0.15, p = 0.001), nickel (r2 = 0.06, p = 0.012), arsenic (r2 = 0.47, p = 0.019) and decreased in manganese (r2 = 0.07, p = 0.011). Population level differences in the Hb values are known^40^. Therefore an analysis of variance for trace metal concentrations by prevalence (incident versus prevalent patients) and sex (male versus female) was performed. As shown in Table 3 no metal by two-way ANOVA was significantly different considering sex alone. Manganese, nickel, cadmium and tin were significantly different as main effects and as previously described with simple t-test. Considering the combination of prevalence and sex, we observed a significant (p < 0.05) difference in the interaction p-value term for zinc; suggesting zinc increases in males and decreases in females. A comparison of mean fold differences for serum trace metal levels in incident and prevalent subject values compared to literature values for upper limit of normal^42–46^ are shown in Fig. 6. As illustrated in Fig. 6, chromium and antimony serum levels were greater than twofold higher that upper limit of normal in both incident and prevalent patients. Significant excursions across the upper limit of normal with dialysis prevalence were observed for the mean vanadium levels (increased) and the mean manganese levels (decreased).

**Table 3:** Main effects and interactions analysis results from the two-way ANOVA test of the trace metal concentrations considering dialysis prevalence (prevalent vs. incident dialysis patients) and sex (male vs. female).

Metal	Two-way ANOVA (main effects, interaction) p values		
	Sex	Prevalence	Sex × prevalence
Mn	0.154	< 0.0001	0.703
Ni	0.124	< 0.0001	0.198
Cu	0.236	0.056	0.958
Zn	0.072	0.678	0.012
Mo	0.490	0.355	0.598
Cd	0.639	0.001	0.431
Sn	0.361	< 0.0001	0.987
Sb	0.546	0.374	0.870
Se	0.774	0.359	0.076
**Logistic regression p value**			
As	0.434	0.449	
V	0.246	< 0.0001	
Cr	0.537	0.057	
Co	0.573	0.329	
Pb	0.936	0.077	

Trace metal symbol definitions provided in methods.

## Discussion

Anemia as well as trace metal derangements are common complications of advanced CKD and ESKD^17,47,48^. Since trace metals are important co-factors in many cellular processes including erythropoiesis and dialysis patients can have altered concentrations of trace metals we looked to see if ESA responsiveness was associated by differing concentrations of metals. We examined at three measures of ESA response: ESA dose, Hb achieved, and a factor that averages these measures over a 6 month period, the ER^49^. Of the trace metals tested, arsenic was the only one to be positively correlated within all three linear regression models for measures of ESA response. Arsenic was infrequently detected in the patient samples and at non-toxic concentrations that approached the maximum contaminant level for drinking water. Arsenic may be encountered in either inorganic or organic forms and with different valence states; arsenites/As(III) and arsenates/As(V)^50^. While our approach did not differentiate the serum samples arsenic valence it is known that arsenic trioxide (arsenite) impacts erythropoiesis and has been used to treat several blood malignancies^51,52^. Zhang et al. have demonstrated a biphasic effect of low dose arsenic trioxide on red blood cell and Hb expression. In studies using CD34 + cells isolated from chronic myeloid leukemia patient bone marrow, sub-toxic levels of arsenic trioxide enhanced or primed erythroid colony forming activity. Additionally the same study demonstrated sub-toxic levels of arsenic trioxide inhibited globin gene expression in K562 cells, a chronic myelogenous leukemia patient derived erythroid-myeloid precursor cell line^53^. While the effect of arsenic appears biphasic in cell culture this cannot be assumed to be the case in the setting of chronic hemodialysis. Hence the positive correlation of arsenic with the anemia end-points (prior month ESA dose, Hb concentration, and ER) may reflect both the combined effects of sub-toxic levels of arsenic and increased erythroid colony formation with increased need for ESA dosing to achieve Hb target levels.

Lead was detected infrequently in patient samples and when observed it was noted the mean concentration decreased from incident to prevalent patients. Similar to arsenic the relative lead concentrations were below what would be considered toxic. Lead was found to be a final component of the linear regression models associated with prior ESA dose and Hb concentration but not ER. Lead is a major public health issue and can cause adverse hematological effects in exposed workers^54^. Lead poisoning interferes with the heme synthesis pathway and can at high enough levels cause iron deficiency ^55^. While there is no safe level of lead in the body, the maximum lead concentration observed in our study was 2.08 µg/dL. This is well below the action level of 5 µg/dL level if detect in children or the concentration of 10–25 µg/dL in adults indicating elevated exposure levels have been encountered. The positive correlation of lead with Hb may reflect ESA dosing overcoming lead impairment of erythropoiesis.

Manganese is an abundant trace metal with many important extra- and intra-cellular functions^56^. Manganese and iron uptake shares a common transporter for dietary uptake (DMT1) and storage (transferrin)^27,57^. Blood manganese and Hb values both positively correlate with renal function^58,59^. Functional iron deficiency defined as insufficient iron incorporation into erythroid precursors despite adequate iron levels is a frequent cause of anemia and often may be associated with high manganese levels^27,60^. Hence the relationship between manganese concentration, Hb and anemia is complicated. Our data showed a significant reduction in manganese from incident (3.0 µg/L) to prevalent (1.1 µg/L) patients. Multivariate linear regression identified six features that best explained the relationship of Hb to observed trace metal concentrations and ESA dose includes manganese. It is difficult to extrapolate the effects of single variables from logistic regression models but these data may point to trace metal interactions that collectively associated with lower Hb values or diminished ER.

The final trace metals found significant in the tests on anemia were cadmium, antimony, vanadium, and chromium. All of these metals are found to be higher in multiple cohorts of dialysis patients ^17,20,61^. Pan et al. report from the National Health and Nutrition Examination Survey (NHANES) data on the association between trace elements (iron, zinc, copper, cadmium, selenium, manganese) and Hb levels in patient with normal renal function or CKD^59^. Regardless of renal function status, Hb concentrations were associated positively with cadmium and negatively with copper. Bayhan et al. report that increased but sub-toxic levels of blood cadmium associate with ineffective erythropoiesis in thalassemia major, thalassemia intermedia and congenital dyserythropoietic anemia ^62^. These data suggest that sub-toxic levels of blood cadmium in individuals with a chronic hypoxic state are susceptible to the contribution of cadmium toward ineffective erythropoiesis.

In our study, cadmium was observed in a majority of patient samples and the median concentrations were higher in prevalent patients. While the cadmium levels were detected below the threshold suggesting toxicity, a regression analysis identified a moderate negative association of cadmium with Hb concentrations (Table 2). Published data would suggest that cadmium could contribute to decreased Hb concentrations either through the development of a state of functional iron deficiency^63^ or direct effects to diminished bone marrow populations of cells in the erythroid lineage, larger circulating populations of immature erythrocytes, and/or smaller populations of mature erythrocytes concomitant with diminished oxygen carrying capacity ^64,65^.

Vanadium showed the greatest accumulation between incident and prevalent patients and between patients on the lowest and highest ESA dose. Vanadium accumulates biologically through inhalation and ingestion as either the vanadate ion (V(V)) or vanadyl ion (V(IV)). Vanadium accumulation in dialysis patient blood has been demonstrated^17,20^. The biochemistry of vanadate and vanadyl ions is complex. The vanadium oxoanions can interfere with the functions of enzymes such as tyrosine kinases, cAMP-dependent kinases, ceramidases, and endothelial cell nitric oxide synthases^5,66–71^. As such, vanadate is commonly included in sample lysis buffer recipes to inhibit tyrosine phosphatases. Collectively the presence of vanadium compounds presence results in altered enzyme function and second messenger signaling^72–74^. It is noteworthy that vanadium compounds are actively taken up by red blood cells to compete with iron binding to hemoglobin^75,76^, influences HIF-1α signaling^68,77^ and also can lead to increased erythrocyte death^78^ with shorten red blood cell life span..

Significant differences exist in trace metal concentrations between incident and prevalent patients (Fig. 5). Vanadium, nickel, cadmium, and tin all appear to accumulate on dialysis while manganese are decreased. Regression analysis demonstrated several trace metals significantly associated with incident and prevalent patients many with low r2 values. These low r2 data suggest that time on dialysis is not the major contributor to the trace metals concentration. While encouraging these observations identify the need for prospective longitudinal studies to define specific trace metal association with dialysis initiation and dialysis vintage that addresses dietary and dialysis fluid trace metal contributions.

The largest changes between incident and prevalent patients were in vanadium and manganese concentrations. The impact of vanadium accumulation on enzymatic processes and the reported relationship between manganese and anemia may explain the observation in the group of patients studied that a greater proportion of subjects requiring an ESA in the prevalent group and that anemia might worsen as dialysis vintage increases. We have observed the increase in ESA requirements in a separate population of patients that were followed over a 4 year period what ESA requirements increased by approximately 400 units/week/year as dialysis vintage increased from 4.6 to 7.3 years^79^.

The current study was developed to examine the association of trace metals and the anemia in hemodialysis patients. Prominent strengths of this study are the prospective recruitment of patients to identify individuals within the first months of dialysis initiation (incident population) as well as chronic dialysis patients. Additional strengths are the use high sensitivity clinical assays that phenotype for important markers of inflammation (hsCRP) and iron availability (hepcidin) and lastly a dialysis population using dynamic ESA dosing protocols delivered to maintain a target Hb value. Limitations of the current study included confounding factors that cannot be addressed due the cross-sectional nature of this study. These confounders significantly included a lack of dietary information for vitamin or mineral supplement usage that might have contributed to observed trace metals, lack of dialysate water sampling, point of source water measurements from patient homes, and due to the study vintage a loss of patients for follow-up due to the low life expectancy of hemodialysis patients. Also the trace metals analysis constrained to a subset of trace metals (n = 14) that did not include some (mercury, strontium, cesium or thallium) previously shown to be associated with death two years after dialysis initiation^3^. Our study examined total serum trace metal levels but not total blood trace metals that would be inclusive of the cellular blood compartment. Some metals exist as organo-metallic compounds (e.g. organo- mercury, lead or tins) such as Sn(II) or the more stable Sn(IV) whose toxicities are associated with the specific organic moiety, or as oxo-acids whose toxicity is associated with a specific oxidation state (e.g. arsenic or chromium)^80–82^. Therefore, absent sample handling to stabilize and separate these charge forms prior to ICP-MS (e.g. metal speciation) we cannot report if the observed associations are with a specific organo-metalloid or charge state (e.g. vanadate versus vanadyl ion). As shown in the Pearson Correlation table (Fig. 1) there are considerable correlations between the trace metals observed such as between nickel positively correlated with tin but negative correlated with copper, selenium, and antimony. Our data provide information for inferring an association of metals with anemia in ESKD but do not shed light on the toxicity mechanism (that of trace metal independent action or concentration addition) or trace metals not measured but correlating with the ones that were measured. Kobayashi et al. demonstrated in a 12-month study of 35 hemodialysis patients the use of zinc supplementation to decrease the need for EPO and decrease in the ER^83^. Lastly, while our study was not designed as an intervention study, our data raise the question of future trials used to examine the concentration addition effects of specific trace metals on ER in ESKD patients.

In conclusion, we report on 14 trace metals and their association with anemia treated with an ESA in both incident and prevalent dialysis patients. Linear regression modeling identified groups of metals that together were significantly associated Hb, ESA dose, and the Hb-dose averaged response of patients (ER). Hb was negatively associated with cadmium while positively associated with antimony arsenic and lead; (b) e amount of ESA dose was negatively associated with vanadium while positively associated with arsenic. The Hb-dose averaged response of patients (‘ER’) to ESAs most strongly positively associated with arsenic. No sex-dependent trace metal differences were observed in incident or prevalent populations. Other observations included significant accumulation of vanadium and reduction in manganese concentration as a function of incident versus prevalent status. These data support future studies for a broader comprehensive metabolomic study into anemia of ESKD and ESA hypo-response with a goal to define targets for interventional studies addressing ESA response and anemia management in hemodialysis populations.

## Methods

### Human subjects

The research protocol conformed to the Declaration of Helsinki and was approved by the Universities of Louisville and Washington Institutional Review Board. Subjects were enrolled following an informed consent process and all data were de-identified. One hundred prevalent (defined as > 6 months on dialysis) and 33 incident (defined as less than 3 months on dialysis) dialysis dependent hemodialysis subjects were enrolled at the University of Washington. Subjects donated 20 mL of blood, processed as both plasma and serum, and stored as 0.5 mL aliquots at − 70∘ until analyzed. These samples were collected using the same protocol and the same serum and plasma collection tubes (BD Vacutainer, Purple top, K3 EDTA 7.0 mL, ref.366450, BD Vacutainer, Red top, serum blood collection tubes, 10.0 mL, ref.366430). Additionally, information on age, gender, race, and hemodialysis vintage as well as up to 6 months of data on EPO (epoetin alfa) dose (units), hemoglobin (Hb, g/dL), ferritin (µg/L), and transferrin saturation (TSAT) were collected.

### Trace elemental analysis

Serum samples (0.5 mL) were digested with a mixture of concentrated nitric acid, hydrochloric acid, and hydrogen peroxide, then analyzed alongside calibration standards, blanks, and appropriate quality control samples to evaluate assay performance. Elements (antimony (Sb), arsenic (As), cadmium (Cd), chromium (Cr), cobalt (Co), copper (Cu), lead (Pb), manganese (Mn), molybdenum (Mo), nickel (Ni), selenium (Se) , tin (Sn), vanadium (V) and zinc (Zn)) were monitored using a Thermo Element 2 high resolution sector field ICP-MS in multiple resolution modes to provide optimal analytical sensitivity and interference resolution for each element. See supplemental information for full method details.

### Hepcidin assay

The hepcidin-25 EIA kit S-1337 was purchased from Peninsula Laboratories (San Carlos, CA). The hepcidin-25 standard was serially diluted. Serum samples were diluted 1:50. Samples and standards were brought to 50 μL and added to an antiserum pre-coated plated for competitive immunoassay according to kit instructions. A uniform volume of 25 μL biotinylated tracer was added to each well of the plate after 2 h and incubated for 1 additional. Captured biotinylated tracer was subsequently bound by streptavidin-conjugated horseradish peroxidase. A color correlated to the sample hepcidin concentrations was produced after TMB substrate was added. The absorbance was read at 450 nm and a four-parameter polynomial regression was used for the standard curve fitting. The coefficient of variation (CV) using a hepcidin-25 concentration of 1.56 ng/mL was 3.49% intra-assay and 3.43% inter-assay. All samples were measured in duplicates and the valid OD was kept correspondent to hepcidin standard between 0.78 ng/mL and 6.25 ng/mL for best reproducibility (lab validation not shown).

### C-reactive protein (CRP) assay

CRP values were determined using the high sensitivity CRP (hsCRP) latex immunoturbidometric assay kit from Roche Diagnostics (Indianapolis, IN). Data were collected on an Roche Diagnostics Cobas Integra 800 using patient serum samples according to the manufacturer’s instructions. Briefly human CRP agglutinates with latex particles coated with monoclonal anti-CRP antibodies. The precipitate is determined turbidimetrically at 552 nm. All samples were measured in duplicates and the valid OD was kept correspondent to the linear range of the hsCRP standards (lab validation not shown).

### Statistical analysis

Statistical analysis was performed using SAS version 9.4 and SPSS version 24. Measured serum concentrations of metals below the limit of detection (LOD) were imputed as ½ LOD if < 25% of the observations were below the LOD and then transformed using the log of the concentration base 2 to address non-normality in the dataset. For metals with a large number of missing values (> 25% of observations below LOD), comparisons of abundances were made based on detection and non-detection events using a chi-square test due to non-normal in the event distribution. Total Epoetin alfa dose (U) was categorized into three groups: 0; 1000 to 29,200; and 30,000 to 136,200. Erythropoietin response (ER) was normalized to achieved hemoglobin over 6 months as ESA Dose/1000 × Hb and categorized into three groups: 0; 0.02 to 2.55; and 2.60 to 11.81. Comparison of means between incident and prevalent hemodialysis groups was performed by t-test for metals < 25% LOD and chi-square for > 25% LOD (chromium, cobalt, lead, arsenic, vanadium). Metals were included in Pearson correlation calculations when observed in at least 75% of all samples. The effects sex and dialysis prevalence was tested using two-way ANOVA to determine main effects and interaction. The effect of metal concentration on erythropoietin response was tested using multivariate regression analysis using backward selection of the metal concentration vs. ESA dose, ER, and current Hb. Regression analysis was performed as linear regression. Post hoc analysis was done by one-way analysis of variance using Student–Newman–Keuls.

## Supplementary information

Supplementary Information.

## Data Availability

Serum ICP-MS and associated data are available in the metabolomics workbench of the NIH Common Fund Metabolomics Program Data Repository and Coordinating Center (DRCC) as study ST000565.

## References

[1] Himmelfarb J, Ikizler TA (2010). Hemodialysis. N. Engl. J. Med..

[2] Saran R (2018). US renal data system 2017 annual data report: Epidemiology of kidney disease in the United States. Am. J. Kidney Dis..

[3] Tonelli M (2018). Concentrations of trace elements and clinical outcomes in hemodialysis patients: A prospective cohort study. Clin. J. Am. Soc. Nephrol..

[4] Thompson S, Tonelli M (2013). Selenium for malnutrition in hemodialysis patients: Have we considered all of the elements?. Nephrol. Dial. Transplant.

[5] Catalan RE, Martinez AM, Aragones MD (1980). Effects of vanadate on the cyclic AMP-protein kinase system in rat liver. Biochem. Biophys. Res. Commun..

[6] Lim HS, Kim HS, Kim JK, Park M, Choi SJ (2019). Nutritional status and dietary management according to hemodialysis duration. Clin. Nutr. Res..

[7] Miskulin D (2009). Key comorbid conditions that are predictive of survival among hemodialysis patients. Clin. J. Am. Soc. Nephrol..

[8] Ikizler TA (2013). Optimal nutrition in hemodialysis patients. Adv. Chronic Kidney Dis..

[9] Sabatino A (2017). Protein-energy wasting and nutritional supplementation in patients with end-stage renal disease on hemodialysis. Clin. Nutr..

[10] Murtas S (2020). Differences and effects of metabolic fate of individual amino acid loss in high-efficiency hemodialysis and hemodiafiltration. J. Renal Nutr..

[11] Suzuki MN (2019). Hemodialysis water parameters as predisposing factors for anemia in patients in dialytic treatment: Application of mixed regression models. Biol. Trace Elem. Res..

[12] Fendley DA, Ward RA (2012). Dialysate quality: New standards require a new approach to compliance. Semin. Dial..

[13] Kalantar-Zadeh K, Kopple JD (2003). Trace elements and vitamins in maintenance dialysis patients. Adv. Renal Replace Ther..

[14] D'Haese PC, De Broe ME (1996). Adequacy of dialysis: Trace elements in dialysis fluids. Nephrol. Dial. Transplant.

[15] Centers for Disease, C. & Prevention. Elevated serum aluminum levels in hemodialysis patients associated with use of electric pumps—Wyoming, 2007. *MMWR. Morbidity Mortality Wkly. Rep.* **57**, 689–691 (2008). 18583956

[16] Guo CH, Wang CL (2011). Plasma aluminum is a risk factor for oxidative stress and inflammation status in hemodialysis patients. Clin. Biochem..

[17] Tonelli M (2009). Trace elements in hemodialysis patients: A systematic review and meta-analysis. BMC Med..

[18] Ari E, Kaya Y, Demir H, Asicioglu E, Keskin S (2011). The correlation of serum trace elements and heavy metals with carotid artery atherosclerosis in maintenance hemodialysis patients. Biol. Trace Elem. Res..

[19] Cross J, Davenport A (2011). Does online hemodiafiltration lead to reduction in trace elements and vitamins?. Hemodial. Int. (Int. Sympos. Home Hemodial.).

[20] Prodanchuk M, Makarov O, Pisarev E, Sheiman B, Kulyzkiy M (2014). Disturbances of trace element metabolism in ESRD patients receiving hemodialysis and hemodiafiltration. Central Eur. J. Urol..

[21] Tonelli M (2015). Trace element supplementation in hemodialysis patients: A randomized controlled trial. BMC Nephrol..

[22] Wang LJ (2017). Effect of zinc supplementation on maintenance hemodialysis patients: A systematic review and meta-analysis of 15 randomized controlled trials. Biomed. Res. Int..

[23] Zhao J, Yan Y, Salnikow K, Kluz T, Costa M (2004). Nickel-induced down-regulation of serpin by hypoxic signaling. Toxicol. Appl. Pharmacol..

[24] Zhao J (2004). Nickel-induced 1,4-alpha-glucan branching enzyme 1 up-regulation via the hypoxic signaling pathway. Toxicol. Appl. Pharmacol..

[25] Deur CJ, Stone MJ, Frenkel EP (1981). Trace metals in hematopoiesis. Am. J. Hematol..

[26] Hoffmeister T (2019). Erythropoietic effects of low-dose cobalt application. Drug Test Anal..

[27] Bjorklund G (2017). Interactions of iron with manganese, zinc, chromium, and selenium as related to prophylaxis and treatment of iron deficiency. J. Trace Elem. Med. Biol..

[28] Garnica AD (1981). Trace metals and hemoglobin metabolism. Ann. Clin. Lab. Sci..

[29] Tanimura, N. *et al.* GATA/heme multi-omics reveals a trace metal-dependent cellular differentiation mechanism. *Dev. Cell* **46**, 581–594 e584, 10.1016/j.devcel.2018.07.022 (2018). 10.1016/j.devcel.2018.07.022 PMC6133724 30122630

[30] Jensen EL (2019). Copper deficiency-induced anemia is caused by a mitochondrial metabolic reprograming in erythropoietic cells. Metallomics.

[31] Beuck S, Schanzer W, Thevis M (2012). Hypoxia-inducible factor stabilizers and other small-molecule erythropoiesis-stimulating agents in current and preventive doping analysis. Drug Test Anal..

[32] Case AJ, Madsen JM, Motto DG, Meyerholz DK, Domann FE (2013). Manganese superoxide dismutase depletion in murine hematopoietic stem cells perturbs iron homeostasis, globin switching, and epigenetic control in erythrocyte precursor cells. Free Radic. Biol. Med..

[33] Feng, H. L., Chen, Y. H. & Jeng, S. S. Effect of zinc supplementation on renal anemia in 5/6-nephrectomized rats and a comparison with treatment with recombinant human erythropoietin. *Int. J. Mol. Sci.* **20**, 10.3390/ijms20204985 (2019). 10.3390/ijms20204985 PMC6829362 31600973

[34] Bradbury BD (2009). Impact of elevated C-reactive protein levels on erythropoiesis-stimulating agent (ESA) dose and responsiveness in hemodialysis patients. Nephrol. Dial. Transplant.

[35] Kalantar-Zadeh K (2009). Predictors of hyporesponsiveness to erythropoiesis-stimulating agents in hemodialysis patients. Am. J. Kidney Dis..

[36] Adamson JW (2009). Hyporesponsiveness to erythropoiesis stimulating agents in chronic kidney disease: The many faces of inflammation. Adv. Chronic Kidney Dis..

[37] Singh AK (2008). The controversy surrounding hemoglobin and erythropoiesis-stimulating agents: What should we do now?. Am. J. Kidney Dis..

[38] Fishbane S, Besarab A (2007). Mechanism of increased mortality risk with erythropoietin treatment to higher hemoglobin targets. Clin. J. Am. Soc. Nephrol..

[39] Murphy WG (2014). The sex difference in haemoglobin levels in adults - Mechanisms, causes, and consequences. Blood Rev..

[40] Lee, G. *et al.* Association of hemoglobin concentration and its change with cardiovascular and all-cause mortality. *J. Am. Heart Assoc.* **7**, 10.1161/JAHA.117.007723 (2018). 10.1161/JAHA.117.007723 PMC5850255 29378732

[41] Merchant ML (2011). Oncostatin M receptor beta and cysteine/histidine-rich 1 are biomarkers of the response to erythropoietin in hemodialysis patients. Kidney Int..

[42] Laboratory, M. C. (2019).

[43] Services, D. O. H. A. H.

[44] Faroon, O. *et al.* in *Toxicological Profile for Cadmium Agency for Toxic Substances and Disease Registry (ATSDR) Toxicological Profiles* (2012). 24049863

[45] Wilbur, S. *et al.* in *Toxicological Profile for Chromium Agency for Toxic Substances and Disease Registry (ATSDR) Toxicological Profiles* (2012). 24049864

[46] Williams, M. *et al.* in *Toxicological Profile for Manganese Agency for Toxic Substances and Disease Registry (ATSDR) Toxicological Profiles* (2012). 24049862

[47] Babitt JL, Lin HY (2012). Mechanisms of anemia in CKD. J. Am. Soc. Nephrol..

[48] Vanholder R, Cornelis R, Dhondt A, Lameire N (2002). The role of trace elements in uraemic toxicity. Nephrol. Dial. Transplant.

[49] Gaweda AE, Aronoff GR, Jacobs AA, Rai SN, Brier ME (2014). Individualized anemia management reduces hemoglobin variability in hemodialysis patients. J. Am. Soc. Nephrol..

[50] Naujokas MF (2013). The broad scope of health effects from chronic arsenic exposure: Update on a worldwide public health problem. Environ. Health Perspect..

[51] Liu JX, Zhou GB, Chen SJ, Chen Z (2012). Arsenic compounds: Revived ancient remedies in the fight against human malignancies. Curr. Opin. Chem. Biol..

[52] Chen SJ (2011). From an old remedy to a magic bullet: Molecular mechanisms underlying the therapeutic effects of arsenic in fighting leukemia. Blood.

[53] Zhang Y (2015). Arsenic primes human bone marrow CD34+ cells for erythroid differentiation. Bioinorg. Chem. Appl..

[54] Goyer RA (1990). Lead toxicity: From overt to subclinical to subtle health effects. Environ. Health Perspect..

[55] Hsieh NH (2017). Anemia risk in relation to lead exposure in lead-related manufacturing. BMC Public Health.

[56] Chen P, Bornhorst J, Aschner M (2018). Manganese metabolism in humans. Front. Biosci. (Landmark Ed).

[57] Fitsanakis VA, Zhang N, Garcia S, Aschner M (2010). Manganese (Mn) and iron (Fe): Interdependency of transport and regulation. Neurotox. Res..

[58] Kim, M., Koh, E. S., Chung, S., Chang, Y. S. & Shin, S. J. Altered metabolism of blood manganese is associated with low levels of hemoglobin in patients with chronic kidney disease. *Nutrients* **9**, 10.3390/nu9111177 (2017). 10.3390/nu9111177 PMC5707649 29077007

[59] Pan CF, Lin CJ, Chen SH, Huang CF, Lee CC (2019). Association between trace element concentrations and anemia in patients with chronic kidney disease: A cross-sectional population-based study. J. Invest. Med..

[60] Kim Y (2005). Blood manganese concentration is elevated in iron deficiency anemia patients, whereas globus pallidus signal intensity is minimally affected. Neurotoxicology.

[61] Palaneeswari, M. S., Rajan, P. M., Silambanan, S. & Jothimalar. Blood arsenic and cadmium concentrations in end-stage renal disease patients who were on maintenance haemodialysis. *J. Clin. Diagn. Res.* **7**, 809–813, 10.7860/JCDR/2013/5351.2945 (2013). 10.7860/JCDR/2013/5351.2945 PMC3681043 23814716

[62] Bayhan T (2017). Heavy metal levels in patients with ineffective erythropoiesis. Transfus Apher. Sci..

[63] Saljooghi AS, Fatemi SJ (2010). Cadmium transport in blood serum. Toxicol. Ind. Health.

[64] Sakata S (1988). Effects of cadmium on in vitro and in vivo erythropoiesis: Erythroid progenitor cells (CFU-E), iron, and erythropoietin in cadmium-induced iron deficiency anemia. Exp. Hematol..

[65] Chatterjee S, Saxena RK (2015). Preferential elimination of older erythrocytes in circulation and depressed bone marrow erythropoietic activity contribute to cadmium induced anemia in mice. PLoS ONE.

[66] Tsiani E, Fantus IG (1997). Vanadium compounds biological actions and potential as pharmacological agents. Trends Endocrinol. Metab..

[67] Tada E (2010). Activation of ceramidase and ceramide kinase by vanadate via a tyrosine kinase-mediated pathway. J. Pharmacol. Sci..

[68] Hwang JT (2004). AMP-activated protein kinase activity is required for vanadate-induced hypoxia-inducible factor 1alpha expression in DU145 cells. Carcinogenesis.

[69] Papapetropoulos A (2004). Vanadate is a potent activator of endothelial nitric-oxide synthase: Evidence for the role of the serine/threonine kinase Akt and the 90-kDa heat shock protein. Mol. Pharmacol..

[70] Daum G, Levkau B, Chamberlain NL, Wang Y, Clowes AW (1998). The mitogen-activated protein kinase pathway contributes to vanadate toxicity in vascular smooth muscle cells. Mol. Cell Biochem..

[71] Klarlund JK, Latini S, Forchhammer J (1988). Numerous proteins phosphorylated on tyrosine and enhanced tyrosine kinase activities in vanadate-treated NIH 3T3 fibroblasts. Biochim. Biophys. Acta.

[72] Ehring GR (2000). Vanadate induces calcium signaling, Ca2+ release-activated Ca2+ channel activation, and gene expression in T lymphocytes and RBL-2H3 mast cells via thiol oxidation. J. Immunol..

[73] Trevino S (2019). Vanadium in biological action: Chemical, pharmacological aspects, and metabolic implications in diabetes mellitus. Biol. Trace Elem. Res..

[74] Huyer G (1997). Mechanism of inhibition of protein-tyrosine phosphatases by vanadate and pervanadate. J. Biol. Chem..

[75] Sanna D, Serra M, Micera G, Garribba E (2014). Interaction of antidiabetic vanadium compounds with hemoglobin and red blood cells and their distribution between plasma and erythrocytes. Inorg. Chem..

[76] Cantley, L. C., Jr. & Aisen, P. The fate of cytoplasmic vanadium. Implications on (NA,K)-ATPase inhibition. *J. Biol. Chem.* **254**, 1781–1784 (1979). 217870

[77] Gao N (2002). Vanadate-induced expression of hypoxia-inducible factor 1 alpha and vascular endothelial growth factor through phosphatidylinositol 3-kinase/Akt pathway and reactive oxygen species. J. Biol. Chem..

[78] Foller M, Sopjani M, Mahmud H, Lang F (2008). Vanadate-induced suicidal erythrocyte death. Kidney Blood Press. Res..

[79] Gaweda AE, Jacobs AA, Aronoff GR, Brier ME (2018). Individualized anemia management in a dialysis facility - Long-term utility as a single-center quality improvement experience. Clin. Nephrol..

[80] Barbosa, C. M. L., Ferrao, F. M. & Graceli, J. B. Organotin compounds toxicity: Focus on kidney. *Front. Endocrinol. (Lausanne)* **9**, 256, 10.3389/fendo.2018.00256 (2018). 10.3389/fendo.2018.00256 PMC5972511 29872423

[81] de Carvalho Oliveira, R. & Santelli, R. E. Occurrence and chemical speciation analysis of organotin compounds in the environment: A review. *Talanta* **82**, 9–24, 10.1016/j.talanta.2010.04.046 (2010). 10.1016/j.talanta.2010.04.046 20685429

[82] Chang LW (1990). The neurotoxicology and pathology of organomercury, organolead, and organotin. J. Toxicol. Sci..

[83] Kobayashi H (2015). Oral zinc supplementation reduces the erythropoietin responsiveness index in patients on hemodialysis. Nutrients.

