# Supplementary material for: Serum trace metal association with response to erythropoiesis stimulating agents in incident and prevalent hemodialysis patients
Source: Sci Rep. 2020 Nov 19;10:20202. doi: 10.1038/s41598-020-77311-8 (PMC7677396; doi:10.1038/s41598-020-77311-8)
Supplement: Supplementary file 1 — Supplementary Information. [file 41598_2020_77311_MOESM1_ESM.pdf]

Serum trace metal association with response to erythropoiesis stimulating agents in incident and prevalent hemodialysis patients.

Michael E. Brier[1,2†] (Ph.D.), Jessica R. Gooding[3,4†] (Ph.D.), James M. Harrington[5] (Ph.D.), Jason P. Burgess[3⁑] (Ph.D.), Susan L. McRitchie[3,4] (M.S.), Xiaolan Zhang[6] (Ph.D.), Brad H. Rovin[6] (M.D.), Jon B. Klein[1,2] (M.D., Ph.D.), Jonathan Himmelfarb[7] (M.D.), Susan J. Sumner, (Ph.D.)[3,4‡], Michael L. Merchant, (Ph.D.)[1,8‡*]

Supplemental Methods

Samples and Materials

Trace metal grade nitric acid was obtained from Fisher Scientific (Pittsburgh, PA) and purified by distillation in a Gateway acid still for use in the digestion process. Concentrated hydrochloric acid (Optima purity) was obtained from Fisher Scientific (Pittsburgh, PA) and used as received for digestion of samples. Hydrogen peroxide (30%, non-stabilized, Suprapur purity, EMD Millipore, Burlington, MA) was also used for the digestion process. National Institute of Standards and Technology (NIST)-traceable 10 mg/L elemental standards were obtained from High Purity Standards (Charleston, SC) for preparation of multielement calibration standards of nickel (Ni), copper (Cu), manganese (Mn), cobalt (Co), chromium (Cr), molybdenum (Mo), selenium (Se) , cadmium (Cd), arsenic (As), lead (Pb), antimony (Sb), tin (Sn), vanadium (V) and zinc (Zn). Single element stock standards of indium (In), praseodymium (Pr), and scandium (Sc) were obtained for use as internal standards. Approximately 18 MΩ cm^{-1} water was used in all sample preparation and analysis steps. NIST standard reference material (SRM) 1598a, Inorganic Constituents in Animal Serum was obtained and prepared alongside study samples to provide a measure of method performance.

Instrumentation

Samples were prepared in a Class 100 clean hood to prevent contamination by atmospheric particulates. Digestion was performed by graphite heating block digestion unit (SCP, Quebec, Canada). After digestion, the samples were analyzed for trace minerals using the Thermo (Bremen, Germany) Element 2 high resolution sector field ICP-MS equipped with a concentric glass nebulizer and Peltier-cooled glass spray chamber.

Digestion method

Prior to digestion, all samples were gently vortexed to provide a homogeneous matrix for digestion. Samples were immediately pipetted to prevent settling prior to removing the sample. A volume of 150 µL of each serum sample was dispensed into an acid-washed plastic digestion tube. Reagent blanks were prepared by addition of deionized water in place of the samples. For each analytical day, seven reagent blanks were prepared to monitor background concentrations of all analytes. In addition, pooled serum samples were prepared with each analytical batch and were compared to indicate reproducibility between analytical days.

To each digestion vessel, 300 µL of concentrated HNO_3, 150 µL of concentrated HCl, and 100 µL of H_2O_2 solution was added. Samples were loosely capped and placed in the graphite digestion block and heated at a temperature of 90 °C for 2 hours. At the end of digestion, all samples were removed from the heating block and allowed to cool to room temperature. In the clean hood, samples were spiked with a multielement internal standard to provide a final concentration of 1.0 ng/mL Sc, In, and Pr, and diluted to the final volume with deionized (DI) H_2O. Samples were stored in a monitored refrigerator at a nominal temperature of 8 °C until analysis.

Elemental Analysis

Samples were analyzed without dilution in the original storage containers to minimize the possibility of contamination. Elements were monitored in multiple resolution modes to provide an optimal combination of sample consumption, sensitivity, and interference resolution. Cd, Co, Cr, Cu, Ni, Mn, Mo, Pb, Sb, Sn, V, and Zn were analyzed in medium resolution mode to resolve polyatomic interferences on many elements. As and Se were analyzed in high resolution mode to allow for resolution of ArCl and Ar_2 interferences, respectively. Cd values were reported for isotope 110 due to high Sn content (>5 ng/mL plasma). Continuing calibration check samples were analyzed at most after every ten samples and consisted of a blank measurement to monitor carryover and a mid-range calibration standard. For the calibration, standards were accepted as passing if their back-calculated concentration was found to be within ±15% of the nominal concentration (±25% of the nominal concentration for the lowest concentration standard). Quality control checks were accepted as passing if the determined concentration for all elements was found to be within ±15% of the nominal concentration. The estimated limit of quantification (ELOQ) was taken as the lowest concentration calibration standard for each analyte and continuing calibration blanks were used to determine limits of detection (LOD) for each analyte element. SRM recoveries of certified and reference elements and spiked pooled serum sample analyte recoveries ranged from 75 to 120% for most elements, indicating favorable method performance.
